# Transposable Elements in TDP-43-Mediated Neurodegenerative Disorders

**DOI:** 10.1371/journal.pone.0044099

**Published:** 2012-09-05

**Authors:** Wanhe Li, Ying Jin, Lisa Prazak, Molly Hammell, Josh Dubnau

**Affiliations:** 1 Graduate Program in Molecular and Cellular Biology, Stony Brook University, Stony Brook, New York, United States of America; 2 Cold Spring Harbor Laboratory, Cold Spring Harbor, New York, United States of America; Thomas Jefferson University, United States of America

## Abstract

Elevated expression of specific transposable elements (TEs) has been observed in several neurodegenerative disorders. TEs also can be active during normal neurogenesis. By mining a series of deep sequencing datasets of protein-RNA interactions and of gene expression profiles, we uncovered extensive binding of TE transcripts to TDP-43, an RNA-binding protein central to amyotrophic lateral sclerosis (ALS) and frontotemporal lobar degeneration (FTLD). Second, we find that association between TDP-43 and many of its TE targets is reduced in FTLD patients. Third, we discovered that a large fraction of the TEs to which TDP-43 binds become de-repressed in mouse TDP-43 disease models. We propose the hypothesis that TE mis-regulation contributes to TDP-43 related neurodegenerative diseases.

## Introduction

Accumulation of TAR DNA-binding protein 43 (TDP-43) containing cytoplasmic inclusions is a shared pathological hallmark in a broad spectrum of neurodegenerative disorders, including ALS, FTLD and Alzheimer's disease [1]. Mutations in this multifunctional RNA binding protein are also known to underlie some familial and sporadic cases of ALS [1]. Despite considerable progress, the mechanisms that link TDP-43 to neurodegeneration still are unclear. We conducted a meta-analysis of TDP-43 protein:RNA target binding datasets and of mRNA expression datasets. All previous analyses of such data focused on sequence reads that uniquely map to the reference genome, thereby excluding transcripts derived from transposable elements (TEs). In contrast, we included sequences that map to multiple locations and examined reads that align to TEs. Our analyses lead to the striking hypothesis that TE over-expression may contribute to TDP-43 mediated neurodegeneration.

Transposable elements (TEs) are highly abundant mobile genetic elements that constitute a large fraction of most eukaryotic genomes. Retrotransposons, which copy themselves through an RNA intermediate, represent approximately 40% of the human genome [2], [3]. Although the majority of TE copies are nonfunctional, a subset have retained the ability to mobilize and even the immobile copies can be expressed [4]. Because of their potential to copy themselves and insert into new genomic locations as well as to generate enormous levels of expression, transposable elements present a massive endogenous reservoir of genomic instability and cellular toxicity [3]. The impacts of these parasitic genetic elements normally are stifled by potent cellular mechanisms involving small interfering RNAs that act via the RNA induced silencing complex (RISC) to inhibit transposon expression ([5] for review). Although most investigations have naturally focused on the germline, where new insertions are heritable and thus favored by transposon evolution, somatic tissues also have an active transposon silencing mechanism whose functional significance is less understood. An emerging literature has established that certain TEs are normally active in brain [6], [7], [8], [9] and elevated expression of some LINE, SINE (which are non-LTR retrotransposons) and LTR elements have been correlated with several neurodegenerative disorders [10], [11], [12], [13], [14], [15], [16]. We therefore investigated whether the RNA targets of TDP-43 include transposon-derived transcripts.

Several recent studies used deep sequencing to profile the RNA targets that co-purify with immunoprecipitated mouse, rat or human TDP-43 and also to profile gene expression changes in mouse after knockdown or over-expression of TDP-43 [17], [18], [19], [20]. In each case, however, these studies analyzed annotated protein coding sequences and excluded TE-derived transcripts and other repetitive elements due to the difficulties inherent in working with ambiguously mapped reads from short read technologies [e.g. [21]]. Despite efforts to develop new algorithms for analyzing multiple alignments of short reads [22], these algorithms have not been applied systematically for analyzing TE-derived transcripts in any neurodegenerative disease. Because each of the above mentioned TDP-43 related studies provided public access to their raw data, we were able to use this resource to search for TDP-43 targets and for transcript mis-expression when we included sequence reads that map to multiple genomic locations, the majority of which are TE derived transcripts in these datasets. Our meta-analysis supports three main conclusions. First, TDP-43 broadly targets TE-derived transcripts, including many SINE, LINE and LTR classes as well as some DNA elements. Second, the association between TDP-43 and TE-derived RNA targets is reduced in FTLD patients relative to healthy subjects, consistent with the idea that loss of TE control might be part of the disease pathology. Third, we observe broad over-expression of TE derived transcripts in each of two different mouse models with TDP-43 dysfunction. Finally there is a striking overlap between the TE transcripts identified as targets and those that are over-expressed with TDP-43 misexpression.

## Results

**Figure 1:**
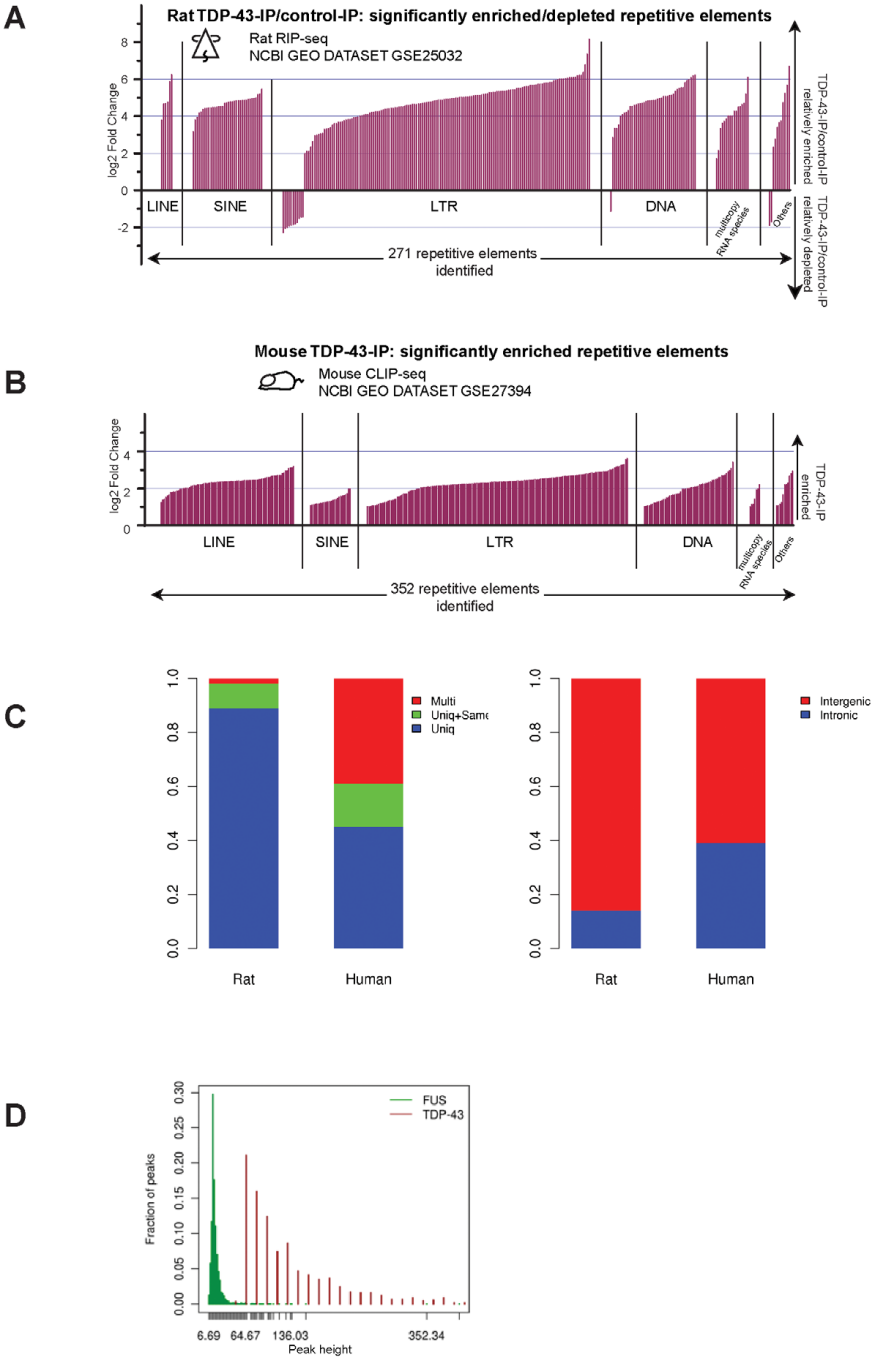
TDP-43 binds broadly to transposable element (TE)-derived transcripts. Magnitude (log2-fold) of enrichments (**up**) or depletions (**down**) are shown (**A**, rat; **B**, mouse) for significantly bound repeat elements grouped by class. **MULTI** method (see text) was used for **A** and **B**. (**C**) The majority of rat TE targets identified with **MULTI** also are identified (**Left Panel, Rat**) when analysis is restricted to reads that map uniquely (**UNIQ**) or when both uniquely mapped and multi-mapped reads that map to the same TE were included (**UNIQ+SameEle**). These conclusions also hold for TE targets whose binding is reduced in FTLD samples from human tissue relative to healthy controls (**Left panel, Human**). Most rat TE targets and differentially bound human TE targets identified from uniquely mapped reads are intergenic (**Right panel**). (**D**) For TDP-43, peaks (UNIQ+SameEle) over TE targets are tall and sharp with mean peak height of 158 counts/peak. In contrast, peak heights are lower for FUS (mean peak height of 17).

We first re-analyzed raw data from the rat TDP-43 RNA immunoprecipitation sequencing (RIP-seq) dataset [17] and the mouse and human TDP-43 *in vivo* crosslinking-immunoprecipitation sequencing (CLIP-seq) datasets [18], [19]. We tested three different analysis methods to examine effects on TEs (Fig. 1A–C; Methods and Figs. S1 and Tables S1, S2, S3). Because reads could potentially map to many regions, we first used an analysis in which each location was weighted based on the number of alignments (Figs. 1A,B) see methods). This analysis method (MULTI), which included both unique and multi mapped reads, assigns an enrichment level for each element, but does not distinguish contributions of individual instances of each element. Although this method can potentially include effects from TEs that are difficult to map with short read sequence, a disadvantage is that it does not distinguish which instances of a given TE are detected. In addition, because many TE copies are present within introns of genes, the MULTI method does not distinguish whether the TE sequences are co-expressed with genes or expressed from TEs *per se*. To address these issues, and to test the robustness of our observations, we also tested two additional mapping methods for the rat and human datasets (Figs. 1C and S1E,F; Methods). First, we examined only the subset of reads that map uniquely to the genome (UNIQ). This method does bias the results to the fraction of TEs that have diverged enough to have unique sequences, but provides confidence that signal derives from unique chromosomal locations. As a third mapping strategy (UNIQ+SameEle), we examined the effects of including both uniquely mapped sequences and those that map to multiple locations so long as they map to the same element (weighted for their contribution to each instance as above – see Methods).

With all three mapping strategies we find a dramatic enrichment of sequences that derive from each major class of TE (Figs. 1A–C; S1; Table S3). With the MULTI method, we find 271 significantly enriched or depleted (most were enriched) repeat element sub-families in the rat TDP-43-IP samples versus control (Fig. 1A), of which 245 correspond to TEs. In the mouse dataset (Fig. 1B), MULTI detects significant enrichment of 352 repeat element sub-families of which 334 correspond to TEs (Table S3). These comprise all major classes of TEs, including LINE, SINE, LTR and some DNA elements [3]. For instance, 85 out of the 122 known mouse LINE elements and 6 out of the 7 known rat LINE elements are identified as TDP-43 targets. Similarly 26 out of 41 mouse SINE elements and 36 out of 37 rat SINE elements also were detected as TDP-43 targets. One caveat to the mouse clip-seq analysis was the lack of a control IP to use in estimating background counts for this single dataset, which could potentially lead to a larger false positive rate in the detected peaks (see Methods); however, the similarity in the results obtained for this dataset as compared to the well-controlled studies for rat (Fig. 1A) and human datasets (see below) argues for the inclusion of this dataset despite its caveats.

Overall, we detect the most extensive binding to TEs with the MULTI method, and these findings are not an artifact of the way we assigned weights with the MULTI method because even with the more restricted UNIQ analysis, we identify ∼80% of the rat elements that are differentially enriched when all mappable reads are included (Figs. 1C, S1F). Moreover, among the uniquely mapped subset of TE instances that we identify as TDP-43 targets, greater than 80% map to intergenic regions rather than to elements contained within genes (Fig. 1C). When we include both unique mappers and multi mappers from the same element (UNIQ+SameEle), we detect enrichment for 95% of the TE sub-families that were identified as TDP-43 targets with the MULTI method (Figs. 1C, S1F). The concordant results from these three different mapping strategies provide confidence that identification of TE derived transcripts as TDP-43 targets is a robust effect that is detected with a variety of methods for dealing with multi-copy elements.

As a test of the biological specificity of our finding that TDP-43 selectively binds to TE derived transcripts, we applied the UNIQ mapping method to a CLIP-seq dataset for an unrelated RNA binding protein. For this purpose we chose fused in sarcoma (FUS), which like TDP-43, is an hnRNP RNA binding protein that plays diverse roles in RNA biology, including splicing [23]. FUS is a relevant control for specificity because like TDP-43, it is implicated in neurodegenerative disorders including ALS [24]. The result with FUS is in stark contrast with TDP-43 (Fig. 1D). For TDP-43, peaks (defined within a 500 bp window) that map to TEs are relatively large, with a mean peak height of 158 counts. In contrast, with FUS we only see small peaks over TEs with a height of just a few counts (mean peak height of 17; Fig. 1D for distribution). Peaks that map over RefGene annotations, on the other hand, are similarly distributed for both FUS and TDP-43 (Mean height of 32 and 68 respectively, Fig. S1H). The distributions of mean peak heights (see histogram, Fig. 1D) shows a clear separation between TDP-43 peaks and those obtained with FUS and this separation between peak heights is statistically significant (Wilcoxon rank sum *p*-value<2.2e^−16^). Thus our findings show specificity for TDP-43 and are not a byproduct of inherent biases in library construction or analysis.

Because TDP-43 has a known binding motif among its mRNA targets, we used MEME ([25] and see Methods) to identify enriched motifs among both the RefGene and repetitive targets. We identify a UGUGU pentamer motif that is equivalently enriched in uniquely mapped and repetitive targets (Fig. S1C; Methods). This motif is consistent with the binding specificity of TDP-43 that has previously been observed for uniquely mapped sequences [17], [18], [19], [20]. Thus TDP-43 binds TE derived transcripts via a similar sequence motif as identified for RefGene targets.

**Figure 2:**
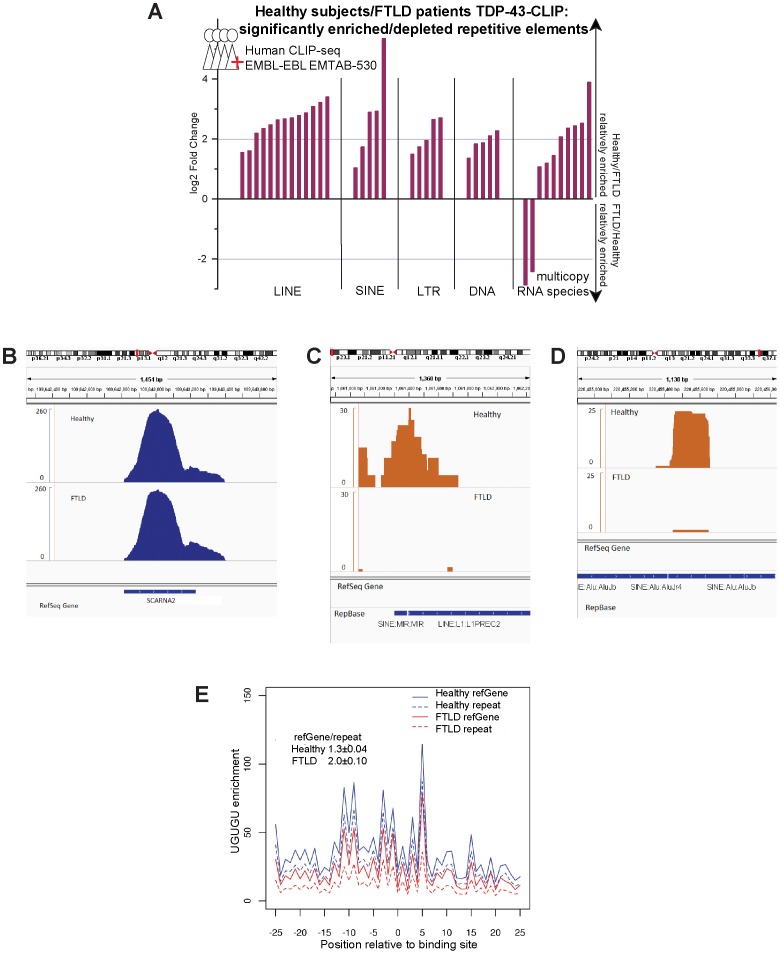
TDP-43 binding to TEs is selectively lost in FTLD patients. (**A**) In the human CLIP-seq data from FTLD versus healthy control, 38 repeat elements showed significant (*p*-value< = 1e-5 and fold changes> = 2) differential binding. Log2 fold binding differences are shown for significantly enriched/depleted elements. (**B,C,D**) Peaks are shown in genome browser for one RefGene control (**B**) and two differentially targeted TEs (**C,D**) in Healthy (**top**) versus FTLD (**bottom**). (**E**) Enrichment for the UGUGU motif relative to its prevalence in the genome is shown across a 51-nt window surrounding binding sites (−25 nt, 25 nt). Healthy samples (**Blue**) show similar enrichment for the UGUGU pentamer motif among RefGene (**solid**) and repeat (**dashed**) sequences (RefGene/repeat motif enrichment ratio ≈1.3). In contrast, motif enrichment in FTLD samples (**Red**) is significantly reduced among repeat (**dashed**) annotations relative to RefGene (**solid**; *p*-value< = 0.01; RefGene/repeat motif enrichment ratio ≈2.0).

Because the human dataset [18] includes samples from healthy and FTLD patients (which exhibit TDP-43 positive cytoplasmic inclusions), it also provided an opportunity to identify differences in the TDP-43 targets between FTLD and healthy controls. As in rat and mouse, we observe in human samples a dramatic and significant enrichment in target sequences that derive from many classes of TEs. As with the mouse and rat data, the distribution of peak heights for TE and RefGene targets of TDP-43 are similar (Fig. S1I), indicating that the targeting of TE transcripts is as robust as it is for RefGene targets. More striking, however, is the comparison between healthy subjects and FTLD patients. When we examine the relative enrichment for each repeat element within healthy vs. FTLD samples, we detect a dramatic difference in binding to TE derived RNAs (Fig. 1E–H). Overall, the association between TDP-43 and TE transcripts is significantly reduced in FTLD patients, which leads to a relative enrichment of 38 repeat elements in healthy versus FTLD, 28 of which correspond to transcripts derived from TEs (Fig. 2 and Table S3; See Methods for statistical analyses). We see reduced binding of TDP-43 to transcripts from all major classes of TE including SINE, LINE, LTR and a few DNA elements. Here too, we observe that the majority of the TE targets whose binding to TDP-43 was reduced in FTLD are consistently identified with all three methods (Fig. 1C). Most of the TE targets that show reduced binding to TDP-43 in FTLD samples are intergenic rather than contained within genes (Fig. 1C). Example peaks are shown for one RefGene control (Fig. 1F) as well as two differentially targeted TEs (Figs. 1G,H).

This reduced binding in FTLD patients of TDP-43 to TE-derived transcripts also is apparent when we examine over-all enrichment for the UGUGU pentamer motif (Figs. 2E and S1) relative to the genome. In the rat and mouse samples as well as in the dataset from healthy human brain samples, we observe equivalent enrichment of UGUGU binding motifs among uniquely mapped (RefGene) versus repetitively mapped (repeat) TDP-43 targets (RefGene/repeat enrichment ratio near 1.0; Fig. S1D; see Methods). In the FTLD-TDP-43-CLIP samples, we also see enrichment for the UGUGU motif among RefGene targets that is equivalent to that seen in healthy subjects (Fig. 2E), but the level of enrichment for this UGUGU motif is significantly lower among the sequences that map to repeat elements. In the FTLD samples, the RefGene/repeat enrichment ratio is increased to 2.0 (Fig. 2E; *p*-value< = 0.01, p-values were assigned with 100 iterations on randomly chosen sets containing 50% of original data; see Methods). In other words, FTLD samples exhibit a selective reduction of binding to TE transcripts and also exhibit reduced UGUGU motif enrichment among the remaining repetitive sequences that still co-purify with TDP-43. This difference in motif enrichment between FTLD and control samples is only manifested among repeat annotations.

The reduced binding of TE transcripts in FTLD patients suggested that TDP-43 pathology might include a loss of TE regulation. We investigated this possibility in two ways. First, we analyzed the repetitive sequence reads from two different mRNA-seq datasets from mouse models of TDP-43 pathology.

**Figure 3:**
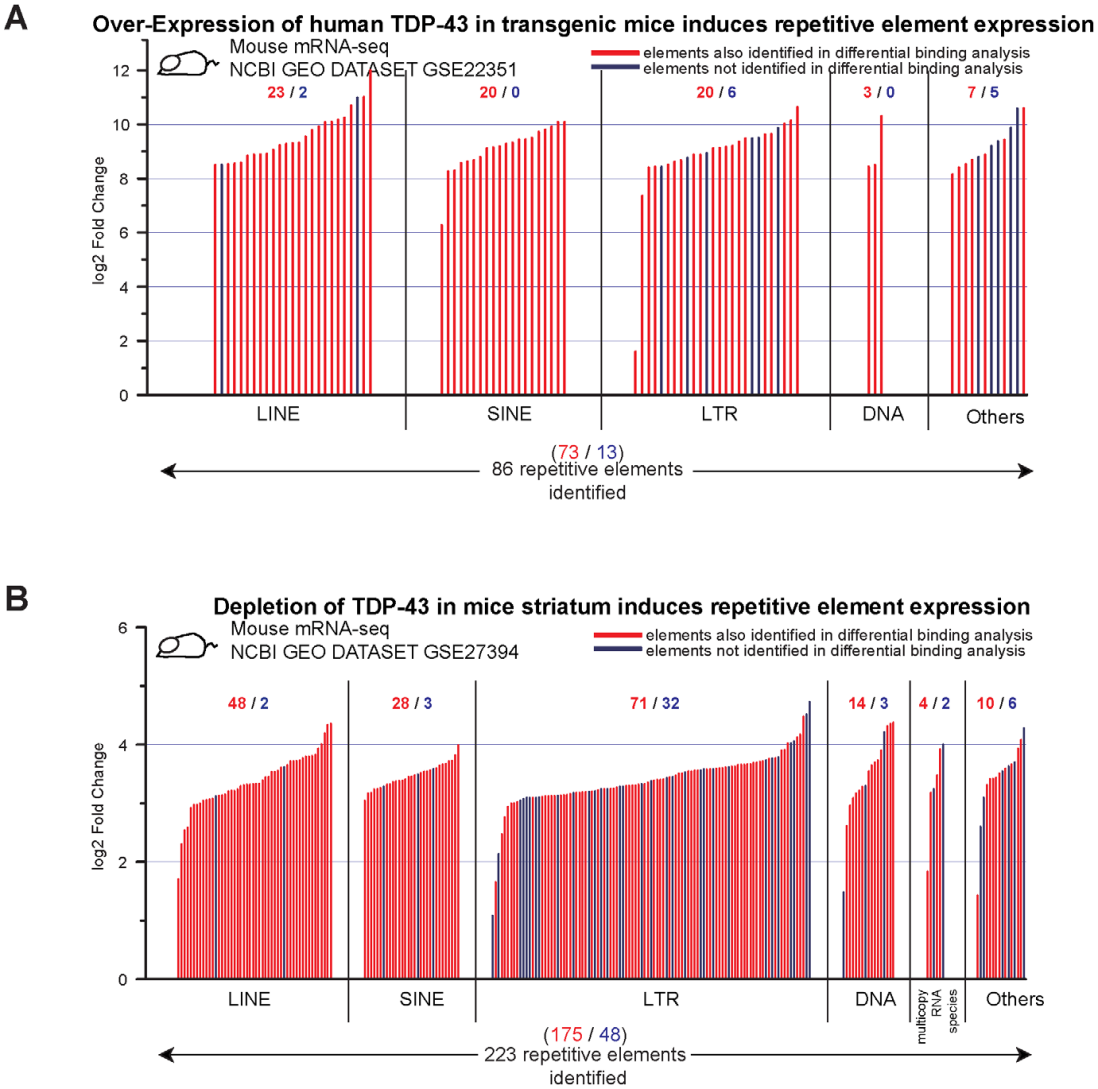
Concordance between mis-regulated TE transcripts upon TDP-43 manipulation and TDP-43 bound TE transcripts. (A,B) Over-expression [20] of TDP-43 in transgenic mice and depletion [19] of TDP-43 in mouse striatum each result in elevated expression of many TE derived transcripts. The majority of over-expressed TEs also were detected (Table S3) as binding targets by CLIP-seq (RED). A few showed elevated expression but were not detected as binding targets (BLUE).

The first mRNA-seq study that we analyzed [20] used over-expression of human TDP-43 in transgenic mice. Overexpression of this aggregation prone protein is associated with toxic TDP-43 pathological effects and is thought to act as a dominant-negative, causing reduction in the normal functions of TDP-43. The second mRNA-seq study [19] used antisense oligonucleotide-mediated depletion of TDP-43 in mouse striatum to test the effects of TDP-43 loss of function. Both studies identified transcripts that are differentially expressed or spliced in response to these TDP-43 manipulations. To ask if the above TDP-43 depletion and over-expression/dominant-negative impacted TE derived transcripts, we again analyzed sequence reads including those that map to multiple locations. We found broad elevations of TE derived transcripts in both the over-expression transgenic mouse model and in the striatal depletion of TDP-43 (Figs. 3A,B). TDP-43 over-expression was associated with elevated expression of 86 repetitive elements (Fig. 3A), whereas TDP-43 depletion results in increased expression levels of 223 repetitive element species (Fig. 3B). In both cases, most of these correspond to LINE, SINE and LTR elements. Overall, the affected TE transcripts are expressed at comparable levels to those of the differentially expressed RefGene transcripts (Fig. S1J), suggesting that these are robust effects on transcripts whose expression levels are not at the limit of detection. More importantly, when TDP-43 function is compromised, we observe a striking degree of concordance between the TE transcripts that are elevated and the ones that we identified as RNA targets of TDP-43 in normal tissue (Red in Fig. 3; See Table S3). Indeed the majority of elevated TE transcripts in both mouse mRNA-seq datasets also were detected as TDP-43 targets in the iCLIP-seq binding dataset (Fig. 3; Table S3). This remarkable concordance between the transcripts that are targeted by TDP-43 and those that are elevated in response to TDP-43 misexpression is unique to the repetitive elements in the genome. In contrast, CLIP targets identified from the RefGene fraction of the transcriptome have little overlap with those that show over-expression when TDP-43 function is compromised suggesting that the coding gene expression increases are largely indirect effects [19]. RefGene transcripts whose expression is reduced show good concordance with direct target identification.

## Discussion

TDP-43 aggregation and neuropathology plays a fundamental role in a broad spectrum of neurodegenerative disorders [1], [26], [27]. This hnRNP-like RNA binding protein already has been implicated in a remarkable number of cellular functions including repression of HIV-1, alternative splicing, regulation of mRNA stability and microRNA biogenesis [26], [27]. Importantly, a large number of cellular targets of TDP-43 have been characterized, leading to the hypothesis that one key role of this multi-functional protein is to regulate alternative splicing of mRNA targets with a preference for those with large UG rich introns [17], [18], [19], [26], [28]. Our findings support the novel hypothesis that TDP-43 also targets the mobile element derived transcriptome. This association is defective in FTLD patients and the TE transcriptome is broadly over-expressed in mouse models of TDP-43 pathology.

A large fraction of the genetic material of multicellular organisms is made up of mobile elements as well as inactivated TEs. A fraction of these TEs retain the capacity to copy themselves and insert at new genomic locations. During the co-evolution of TEs with their host genomes, organisms have evolved elaborate and efficient mechanisms to prevent or at least regulate such transposition events. As a result, even the potentially active TE copies rarely mobilize within the germline and are also largely constrained in somatic tissue. Several recent studies demonstrate, however, that LINE-1 elements are normally active and mobile during neurogenesis in both rodent and human tissue [7], [8], [9]. Somatic mobilization of Alu and SVA elements as well as LINEs also has recently been detected in several different human brain regions [6]. This raises the intriguing hypothesis that active mobilization of some TEs plays a role in normal brain development or physiology. On the other hand, there also is emerging evidence that unregulated activation of TEs is associated with neuropathology. TE activation in brain has been observed in macular degeneration [14], Rett syndrome [11], Prion diseases [13], [29], Fragile-X associated tremor/ataxia syndrome (FXTAS) [15] and ALS [12]. Moreover, for the cases of macular degeneration and FXTAS, there is evidence that activation of SINEs and an LTR-retrotransposon respectively may contribute to the observed pathology [14], [15].

Our findings support three conclusions. First, that TDP-43 broadly targets TE-derived transcripts, including many SINE, LINE and LTR classes as well as some DNA elements. This conclusion is replicated in three independent datasets from rat, mouse and human. Second, the association between TDP-43 and TE-derived RNA targets is reduced in FTLD patients relative to healthy subjects, consistent with the idea that loss of TE control might be part of the disease pathology. Third, we observe broad over-expression of TE derived transcripts in each of two different mouse models with TDP-43 dysfunction. And there is a striking overlap between the TE targets identified in the CLIP study and those that are over-expressed with TDP-43 misexpression. Taken together, our findings raise the hypothesis that TDP-43 normally functions to silence or regulate TE expression. When TDP-43 protein function is compromised, TEs become over-expressed. Unregulated TE expression can have a number of detrimental impacts including genome instability, activation of DNA-damage stress response or toxic effects from accumulation of TE-derived RNAs or proteins. Such toxicity from activation of mobile genetic elements may contribute to TDP-43-mediated neurodegenerative disorders.

## Methods

### Data preparation

The CLIP-seq data of human healthy and FTLD brain tissues was obtained from EMBL-EBL Array Express Archive EMTAB-530 [18]. The RIP-seq data of rat cortical neuron cells was obtained from NCBI GEO DATASET GSE25032 [17]. The mouse CLIP-seq and mRNA-seq datasets were obtained from NCBI GEO DATASET GSE22351 and GSE27394 [19], [20]. The FUS PAR-CLIP-seq dataset [23] was downloaded from DDBJ Sequence Read Archive (DRA) SRA025082. The genome sequences (build rn4, hg19, and mm9), RefGene annotations, and coordinates of repetitive elements in the whole genome of rat and human were downloaded from the University of California, Santa Crutz (UCSC) Genome Browser [30]. Annotation strategies for identified peaks are described in more detail below.

### Alignment

We used Bowtie [31] version 0.12.7 to align the short sequences. Rat and human genome sequences were downloaded from the University of California, Santa Crutz (UCSC) Genome Browser [30]. Two mismatches in the first 25 bp were allowed and the best alignments were reported. For non-uniquely mapped reads, allowing all possible alignments resulted in some reads that could potentially map to more than 10,000 regions. To capture the reads mapped to repetitive regions as much as possible while reducing the space and computational (time) cost, we set the –m option (reported number of alignments per sequence) to a value such that at least 90% of the reads with multiple alignments were reported. Specifically, -m 100, -m 500 and -m 200 were used on rat, mouse and human samples, respectively (command line e.g., -n 2 -l 25 -a -m 100 –best –strata). Each alignment was then assigned a weight in a way that the total weight of all reported alignments of each mapped read is the same. For example, if a read x uniquely maps to a region, then the weight of this alignment is 1. If a read y maps to two regions with the same quality, then each alignment y1 and y2 has weight 0.5, such that the total weight of y is 1. These weights were uniform among the alignments, and did not include a contribution from mapping quality scores because only equivalently mapped alignments were reported (i.e., the “–best –strata” options in the above command line). Table S1 summarizes the mapping results. The FUS dataset had shorter read lengths (36 nt) and lower sequencing qualities than the TDP-43 datasets. For this dataset, we used reads at least 18 nt in length after removing adapters and trimming the last few bases that have low qualities. About 25% of the remaining reads from the FUS dataset mapped uniquely. Finally, prior to normalization and peak identification, presumptive PCR duplicates were removed. For the human CLIP-seq datasets, where randomized nucleotides were included in the sample barcodes, PCR duplicates were identified directly and removed. For all other datasets, PCR duplicates were identified using the Picard “mark duplicates” task and removed prior to further analysis. While the reads in these samples were strand specific, we allowed the reads to map both sense and anti-sense to the Refseq and UCSC annotated gene and TE transcripts. While 98.5% of the reads that derive from Refseq transcripts mapped in the same orientation as the annotated gene, surprisingly, only 50% of the TE-mapped reads mapped to the annotated strand of the TE locus. This was true for both uniquely mapped reads as well as reads mapped to multiple loci.

### Normalization

We chose a bin correlation approach as described in PeakSeq [32] to normalize the libraries, after comparing it with the most widely used library size normalization method. Figure S1A shows the comparison of the predicted differentially bound repeat elements. The bin correlation approach turned out to be more conservative than the library size method. The main reason is that in the control-IP sample, the total number of aligned reads is dominated by a few regions, mostly rRNA repeats, such that using library sizes as a normalization factor will cause a bias towards non-rRNA repeat regions in TDP-43-IP samples. To compute the bin correlation, the whole genome was separated into adjacent non-overlapping 10 Kbp bins. Then the number of reads overlapping with each bin was calculated for all libraries. Notice that each read (alignment) will only be counted once, and the count here is actually the weight of the alignment. Suppose that three reads with alignment weight 0.5, 1, and 0.5 fall in a bin b, then the count of b is 2 instead of 3. The library with the largest number of mapped reads was chosen as a reference. A linear regression was applied to bins of every other library against those of the reference. The correlation coefficient was used as the normalization factor, i.e., L_i_≈e_i_ * L_r_ where *L_r_* is the reference library, *L_i_* is one other library, and *e_i_* is the correlation coefficient of library *L_i_* to *L_r_*. Figure S1B shows the distributions of weighted bin counts between control-IP and TDP-43-IP samples from rat. The majority of bins with high values in either library show large differences, and these bins probably contain the true differential binding sites. These were excluded from the normalization procedure, and only the low abundance bins, colored red in Figure S1B were used to estimate the background for library normalization. The underlying assumption is that the background of the two libraries is similar.

### Differential binding analysis

To identify potential differential binding sites of TDP-43, a sliding window with size of 500 bp and moving step size of 100 bp was used to scan the genome and compute the number of reads falling in the window in both samples. The reason for partially overlapping windows is to increase the resolution at which optimal peaks can be discovered. As described above, the counts in each bin are weighted by the number of loci to which they were mapped. For the rat data, the read counts were modeled with a Poisson distribution, similarly to two popular ChIP-seq analysis approaches, MACS [33] and PeakSeq [32]. In the case of human data, in which each treatment has 3 biological replicates, an over-dispersed Poisson distribution (negative binomial distribution) was applied to model the read counts. In both cases, the *p*-value of the difference of the read counts was calculated as described in DESeq [34]. Given a window *w_i_* with reads *k_iA_* and *k_iB_* from libraries A and B, and *k_iA_*+*k_iB_* = *k_iS_*, the *p*-value of (*k_iA_*, *k_iB_*) is the probabilities of all pairs with probabilities less than or equal to *p*(*k_iA_*, *k_iB_*) among all combinations, i.e.,
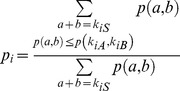
where *p*(*a*, *b*) is *p*(*a*)*p(*b*), by assuming the two libraries are independent, and *p*(*x*) was computed using either the Poisson distribution or a negative binomial distribution. The null hypothesis we are testing against with a negative binomial model states that it is statistically unlikely for a combination of (1) random selection of transcripts sequenced and (2) biological variation between replicates to create a differential enrichment of reads within the given window that is larger than what we see in the TDP-43 IP data as compared to the control. For the rat samples, which did not include replicates, we can only test against a Poisson model null hypothesis that random selection of sequenced transcripts would be statistically unlikely to result in differential enrichment greater than what is seen in the data. These p-values were corrected for multiple hypotheses testing using the Benjamini-Hochberg correction. We set a significance threshold, adjusted *p*-value<0.00001, for identifying differentially enriched regions. We next advanced the sliding window by 100 bp and repeated the previous step. Enriched regions with a gap of less than 500 bp and with the same direction for differential enrichment (i.e., both TDP-43 enriched or both depleted) were merged.

The above differential binding analysis method was used to analyze the datasets in three different ways. For the UNIQ method (see text), only uniquely mapped reads were included. For the UNIQ+SameEle method (see text), unique reads and those that mapped to multiple locations were included, so long as they mapped to the same element. For MULTI (see text), we included all mappable reads.

### Annotation

A predicted region was annotated as ‘RefGene’, if it overlaps with exons of a gene, or as ‘repeat’ if it overlaps with a repetitive element. If a differential binding site overlaps with a repeat region, but this repeat region is inside an exon, then the region will be annotated as the corresponding gene. Simple repeats that overlap with other repeat classes are not considered. The annotations were obtained from the UCSC genome website, as described above, which provides 4 levels of classification for most repeat elements: Class, Family, Element, and Instance. This nomenclature approximates that used by the RepBase group, from which these annotations were derived [35]. An example of that annotation information would be: Class I (retrotransposons), LINE/L2, L2b, chr1:23803–24038. Any cross-comparisons between datasets and species took place at the “Element” level (L2b in the above example), since TE instances (loci) are usually not conserved across evolutionarily distant species and, for the case of the loci that included multi-mapping reads, unambiguous identification of the particular locus from which the reads derived was difficult for many instances.

### Motif Enrichment analysis

MEME [25] was used to identify the most enriched motifs of the TDP-43 binding sequences at repetitive regions, shown in Figure S1C. Both the distribution of each single nucleotide and dinucleotide were computed and used as the MEME background model. Analysis of the most enriched pentamer, UGUGU [18], on both genes and repetitive regions was performed in a similar way as described [36]. The number of reads containing the pentamer at each nucleotide position surrounding the binding sites in a range of [−25 nt, 25 nt] was calculated and then normalized against randomized data. The control data (random data) was generated 100 times with randomly selected binding position sites. To test the robustness of the enrichment difference in the library from subjects with FTLD samples, we did random samplings in two ways. a) Random samples of the healthy brain subjects were selected 100 times, to look for differential enrichment of the UGUGU motif among sub-samples of the healthy peaks. None of them show such a dip in motif enrichment. b) We also randomly selected 50% of the peaks from the healthy and FTLD brains and tested RefGene/repeat motif enrichment ratios in these sub-samples to estimate the sampling error on the estimated RefGene/repeat motif enrichment ratios.

### Binding site identification from mouse CLIP-seq data

The approach described above is not suitable to the mouse dataset, because of a lack of control samples. Therefore, a similar method [37] as used by the authors of the dataset was applied here. As a control, CLIP reads were randomly assigned to genes and annotated repetitive regions. The significance of the cross-link sites were computed by comparing the observed probability of the abundance (cDNA counts) to the background frequency. The background frequency was obtained by iterating the randomization 100 times. The adjusted *p*-value for a cross-link site with cDNA counts *x* was computed as *p^adj^*(*x*) = (*μ_x_*+*σ_x_*)/*p_x_*, where *μ_x_* and *σ_x_* are the mean and standard deviation of frequency of cDNA counts *x* in the randomized background across 100 iterations, and *p_x_* is the observed probability. This method is not as robust as that used for the rat and human peak identification due to the non-random rates of transcription in the genome. The *p*-values shown in Table S3 reflect confidence that candidate binding sites are significant with respect to a model in which reads are otherwise randomly distributed genome-wide. Such a background model is known to be false for gene transcripts, but it is unclear the extent to which this model would fail for transcripts derived from repetitive element loci. At any rate, the lack of a control sample constrains our ability to estimate the background accurately for this single dataset.

### mRNA-seq analysis

RNA short sequences were aligned to the whole genome in order to assess the RNA profiles of repetitive elements. The alignment software and most of the parameter settings were the same as that used for aligning the CLIP-seq datasets (described above, except -m 200 was used in this case). The same weighting scheme was applied to each alignment as described above. Read abundances of a repeat element were computed by summing up the alignment weight of all reads mapped to the correct strand, within the TE annotation boundaries, and normalized by the length of that element. DESeq [34] was then used to detect differential abundances for repeat elements between control and TDP-43 manipulated samples.

## Supporting Information

Figure S1 **Additional bioinformatics analyses.** (**A**) Total candidate differentially enriched peaks annotated as transposable elements (TEs) found using two normalization methods for the Rat TDP-43-IP samples. The left panel shows overlap in the Rat TDP-43 total number of enriched repetitive element TE peaks identified using the two normalization methods, the right panel shows overlap in the number of candidate depleted repetitive element TE peaks. In both orange circles represent (to scale) the number of differential TEs identified when a “bin correlation” approach is used to normalize the reads in each sample, while the blue circles represent the differential TEs using a “library size” normalization approach. The library size normalization approach, which is commonly used, simply normalizes all samples by the total mapped mass of reads in each sample (i.e., reads per million mapped, or RPM); the underlying assumption would be that the background is approximately the same for both samples genome-wide. We noticed that the backgrounds of the control and TDP-43-IP samples were highly non-random, and that some regions had much higher or lower reads than other genomic loci, even outside of the identified binding peaks. Therefore, we modeled the background using a sliding window of non-overlapping 10 kb bins, computing the correlation coefficient between the control and IP samples in each bin, e.g., a “bin correlation” approach to normalization. As is evident from the Venn diagrams in this figure, this approach is more conservative than a simple RPM or “library size” normalization method (please see Methods and Fig. S1B for additional details). (**B**) The whole genome was separated into non-overlapping adjacent 10 Kbp bins. Each dot (black) represents read counts of a bin. Those bins selected to compute the normalization factors were colored in red. (left) Read counts of TDP-43-IP sample and control-IP sample from Rat RIP-seq (right) Read counts of two human healthy brain samples from CLIP-seq data. (**C**) Motif logos for the most enriched motifs as identified by MEME in the TDP-43 binding peaks overlapping repetitive regions. (top) Rat RIP-seq data (bottom) Human CLIP-seq healthy brain tissue samples. (**D**) Enrichment for the UGUGU pentamer motif across a 51 nt window surrounding the binding site (−25 nt, 25 nt) relative to the genome is shown among RefGene and repeat sequences. Mouse (top panel) and rat (bottom panel). (**E**) For each read having multiple alignments (multi-read), the fraction of the most frequently appearing TE among all those alignments is computed. And the distribution of all multi-reads with different common TE alignment fractions is computed. For about 80% of the multi-reads, all alignments corresponded to the same TE element in Rat (top panel) and about 50% in Human (bottom panel). (**F**) Overlaps of detected TEs with each of three mapping methods for Rat (left panel) and Human (right panel) are shown. The three mapping methods are: UNIQ (uniquely mapped reads), UNIQ+SameEle (uniquely mapped reads and multi-reads mapped to the same elements), and MULTI (unique reads and multi-reads). (**G**) Extensive overlap is observed between TE transcripts that were de-repressed with over-expression [1] or depletion [2] of TDP-43 in mouse (Top). Far less concordance is seen with RefGene targets and RefGene transcripts that were over-expressed (bottom). It should be noted however that good correspondence is seen between TDP-43 RefGene targets with long introns and those whose expression is decreased [1]. (**H**) For TDP-43 and FUS, distributions of peaks (UNIQ+SameEle) over RefGene targets are not significantly different from each other. Mean peak heights of TDP-43 and FUS are 68 and 32 respectively. The distance between TDP-43 and FUS is less than 15 with a *p*-value of 0.98. (**I**) For RefGene and repeat sequences that bind to TDP-43 in tissue from healthy human subjects, distributions of peak heights are not significantly different from each other. (**J**) For Refgene and repeat sequences from the mouse TDP-43 overexpression dataset, distributions of expression levels are not significantly different from each other.
(DOCX)

Table S1 **Number of aligned reads for each TDP-43 dataset.** Human dataset is from EMBL-EBL ArrayExpress Archive EMTAB-530 and rat dataset is from NCBI GEO DATASET Accession Number: GSE25032. The mouse datasets are from NCBI GEO DATASET Accession Numbers: GSE22351 and GSE27394.
(DOCX)

Table S2 **Number of aligned reads for FUS datasets.** FUS dataset is from DDBJ Sequence Read Archive (DRA) Accession Number: SRA025082.
(DOCX)

Table S3 Enriched and depleted repetitive elements rat TDP-43-IP/control-IP RIP-seq. Enriched and depleted repetitive elements in Human TDP-43 Healthy/FTLD CLIP-seq. Enriched repetitive elements in mouse TDP-43 CLIP-seq. Induced repetitive elements expression after depletion of TDP-43 with ASO (mouse mRNA-seq). (overlap with mouse CLIP binding sites shown). Induced repetitive elements expression in transgenic mice overexpressing human TDP-43 (mouse mRNA-seq). (overlap with mouse CLIP binding sites shown).
(XLS)
