# Supplementary material for: Transposable Elements in TDP-43-Mediated Neurodegenerative Disorders
Source: PLoS One. 2012 Sep 5;7(9):e44099. doi: 10.1371/journal.pone.0044099 (PMC3434193; doi:10.1371/journal.pone.0044099)
Supplement: Figure S1 — Additional bioinformatics analyses. (A) Total candidate differentially enriched peaks annotated as transposable elements (TEs) found using two normalization methods for the Rat TDP-43-IP samples. The left panel shows overlap in the Rat TDP-43 total number of enriched repetitive element TE peaks identified using the two normalization methods, the right panel shows overlap in the number of candidate depleted repetitive element TE peaks. In both orange circles represent (to scale) the number of differential TEs identified when a “bin correlation” approach is used to normalize the reads in each sample, while the blue circles represent the differential TEs using a “library size” normalization approach. The library size normalization approach, which is commonly used, simply normalizes all samples by the total mapped mass of reads in each sample (i.e., reads per million mapped, or RPM); the underlying assumption would be that the background is approximately the same for both samples genome-wide. We noticed that the backgrounds of the control and TDP-43-IP samples were highly non-random, and that some regions had much higher or lower reads than other genomic loci, even outside of the identified binding peaks. Therefore, we modeled the background using a sliding window of non-overlapping 10 kb bins, computing the correlation coefficient between the control and IP samples in each bin, e.g., a “bin correlation” approach to normalization. As is evident from the Venn diagrams in this figure, this approach is more conservative than a simple RPM or “library size” normalization method (please see Methods and Fig. S1B for additional details). (B) The whole genome was separated into non-overlapping adjacent 10 Kbp bins. Each dot (black) represents read counts of a bin. Those bins selected to compute the normalization factors were colored in red. (left) Read counts of TDP-43-IP sample and control-IP sample from Rat RIP-seq (right) Read counts of two human healthy brain samples from CL [file pone.0044099.s001.docx]

**Figure S1**

**Figure S1A.**

Bin correlation

Library size

Library size

Bin correlation

**Figure S1B.**


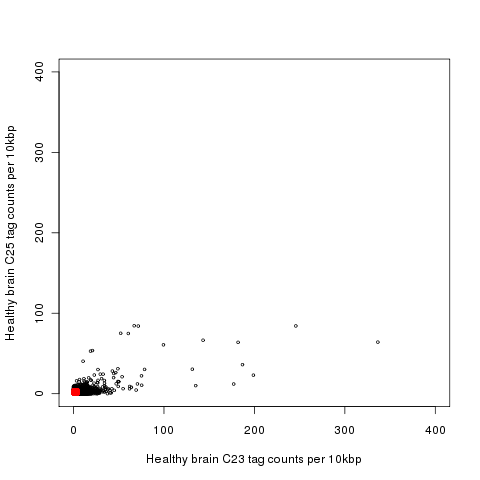

**Figure S1C.**

**
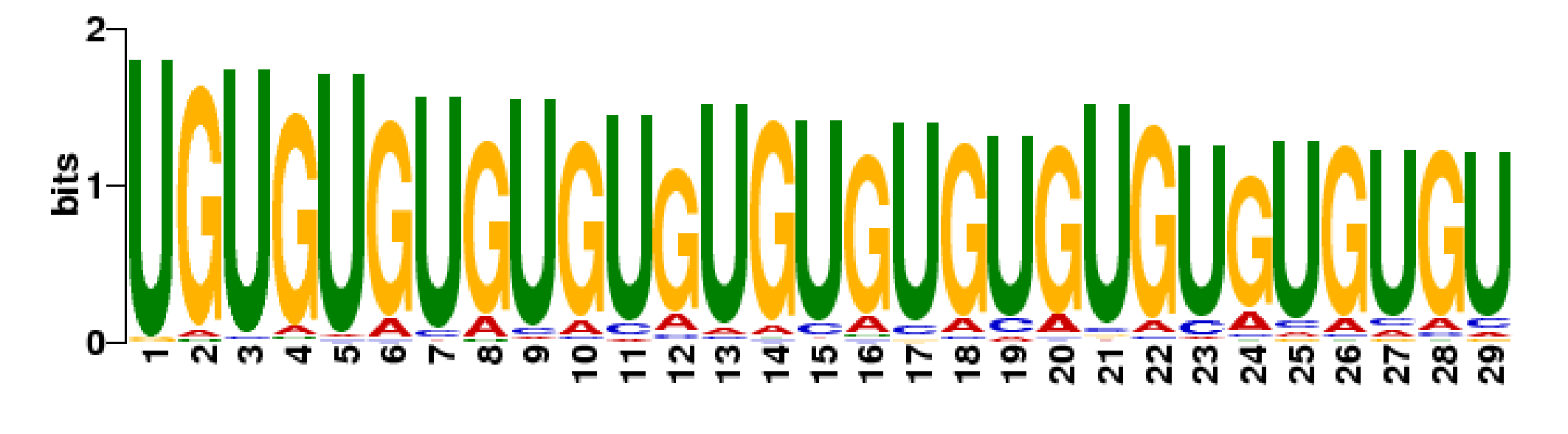
**


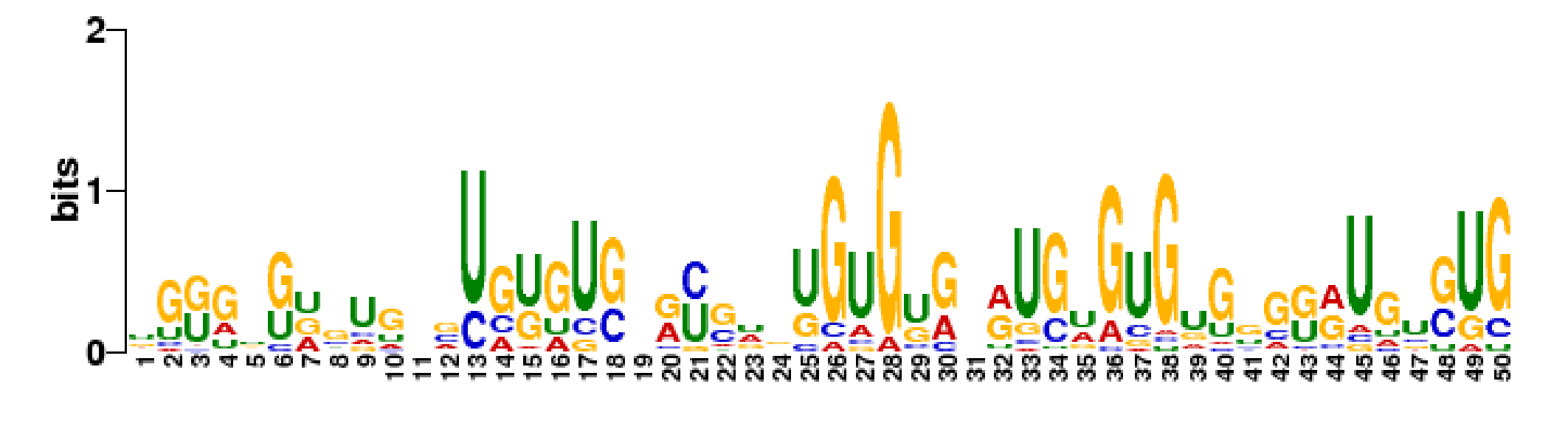


**Figure S1D.**


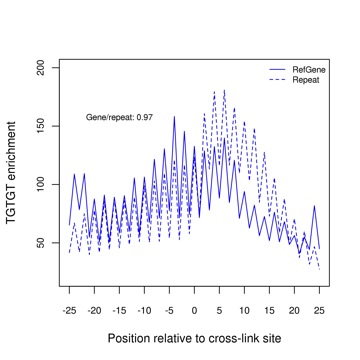

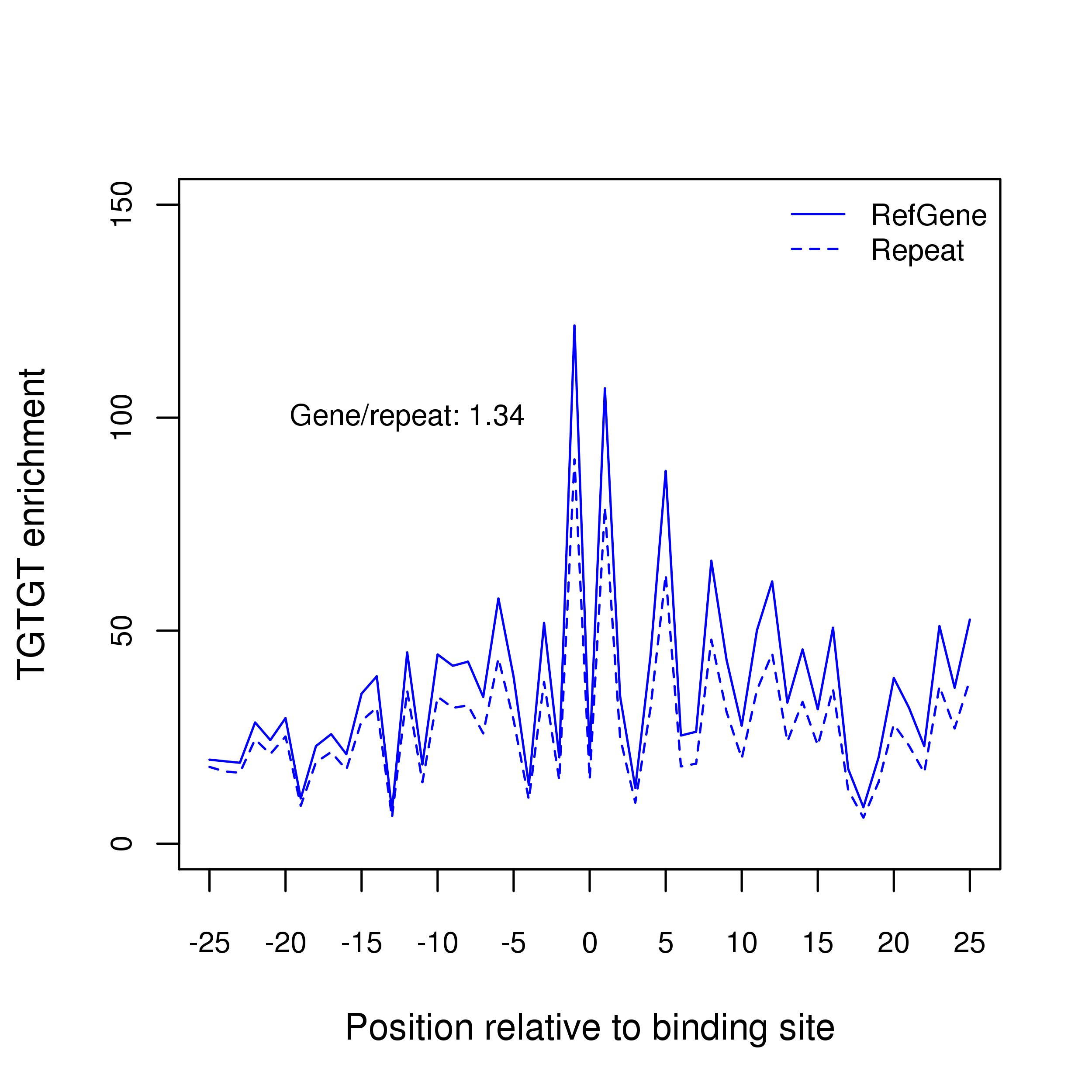


**Figure S1E.**


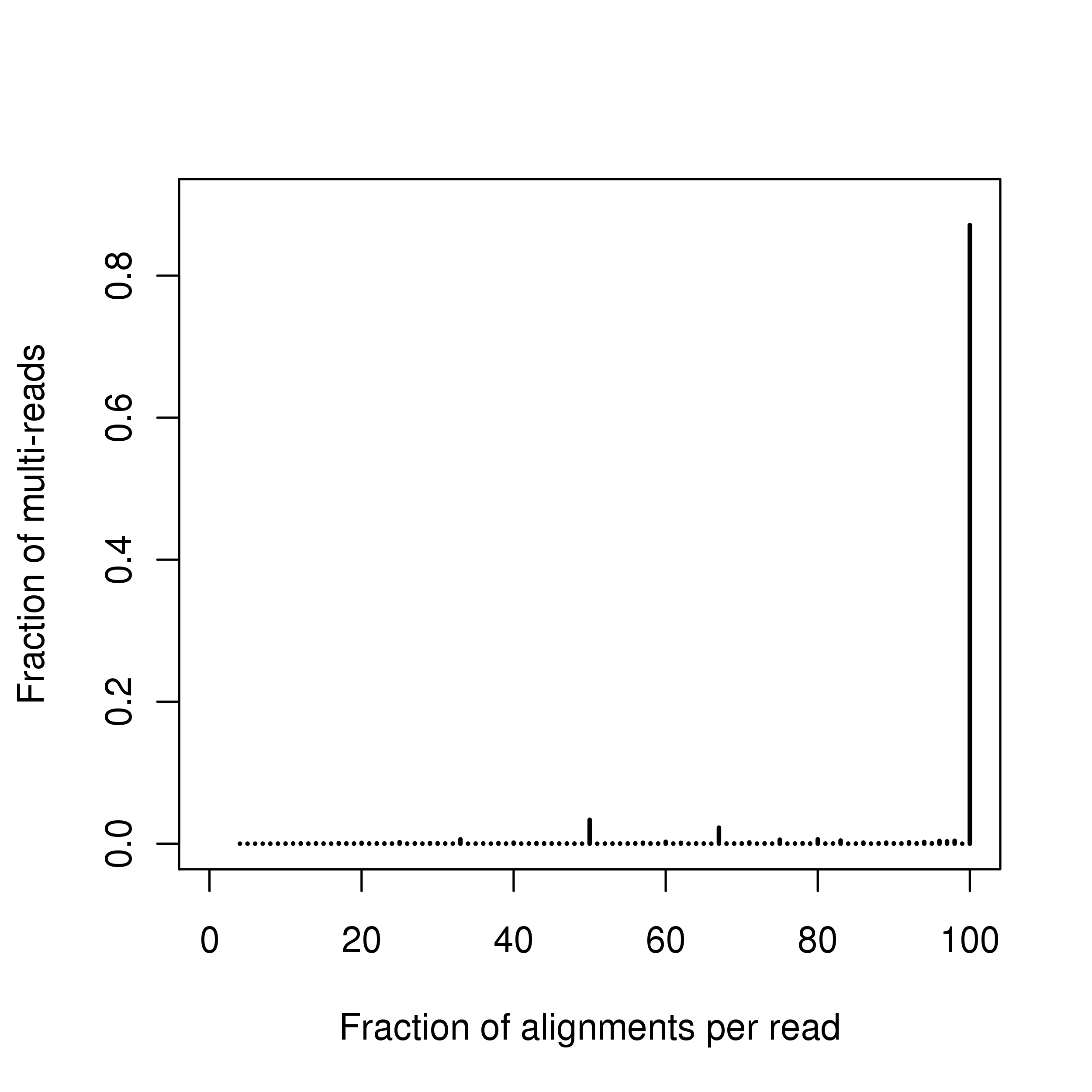


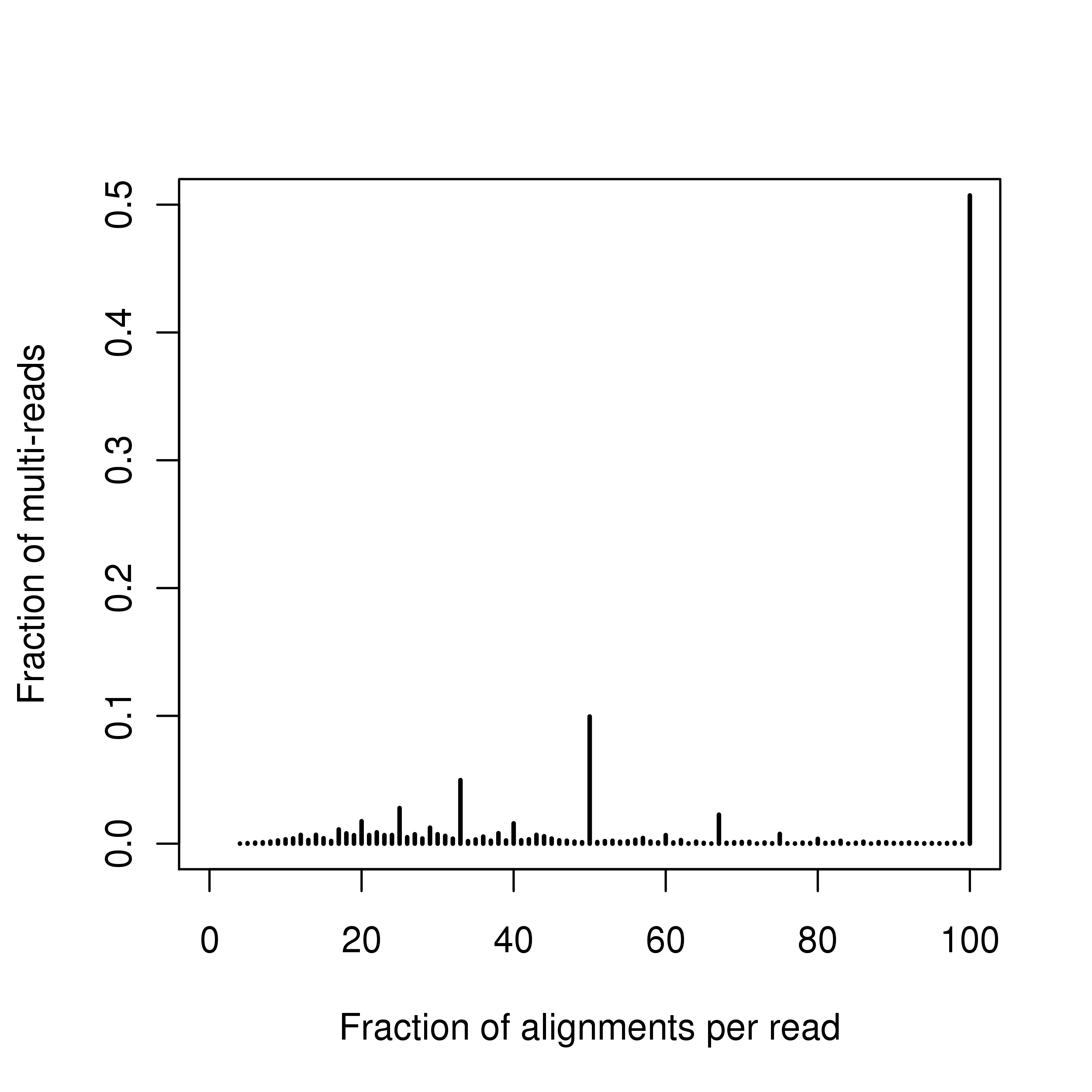


**
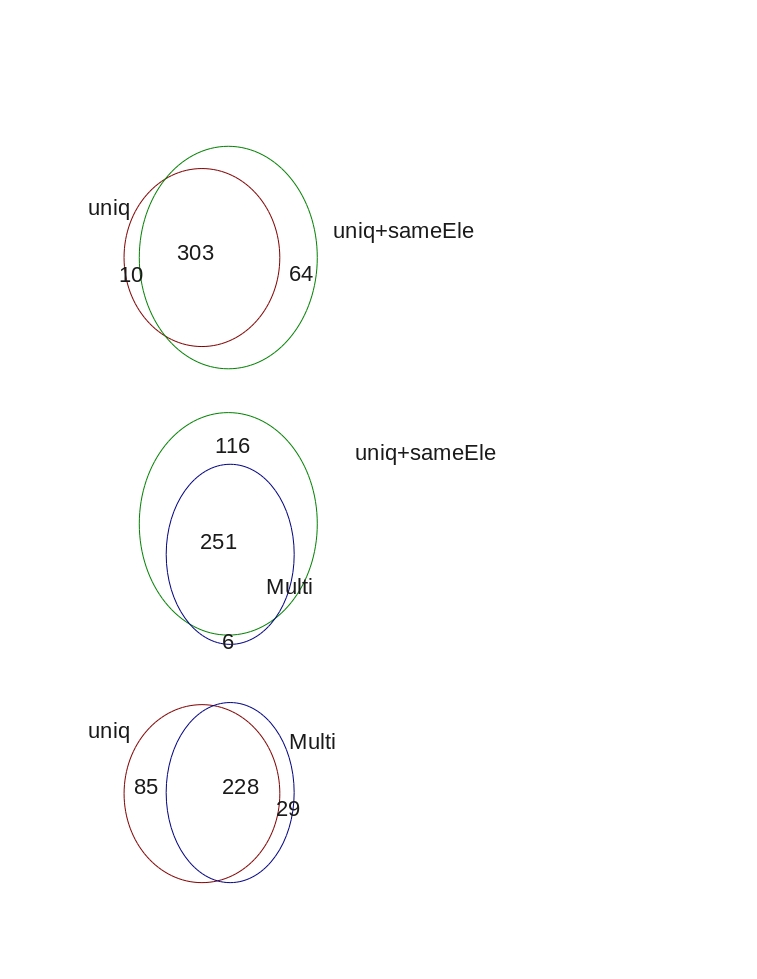

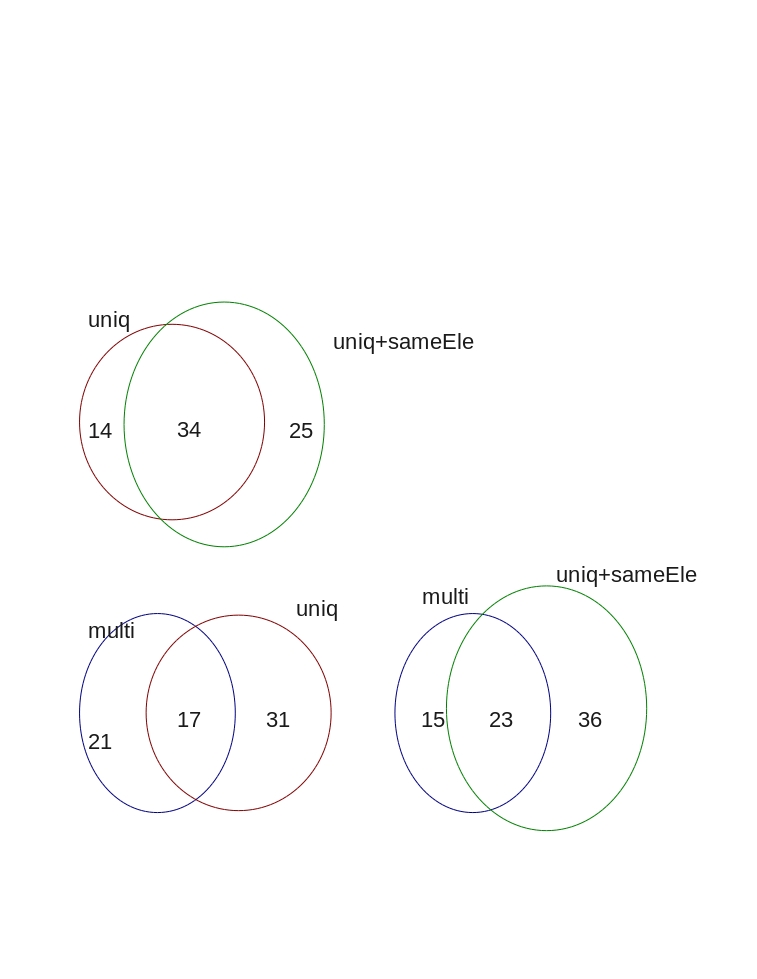
Figure S1F.**

**Figure S1G.**

Over-expression (mRNA-seq)

197

Binding sites (CLIP-seq)

175

48

Overlap of binding sites vs. over-expression on **TE transcripts** in mouse dataset GSE27394.

1201

167

17

Binding sites (CLIP-seq)

Over expression (mRNA-seq)

Overlap of binding sites vs. over-expression on **RefGene transcripts** in mouse dataset GSE27394

**Figure S1H.**

**
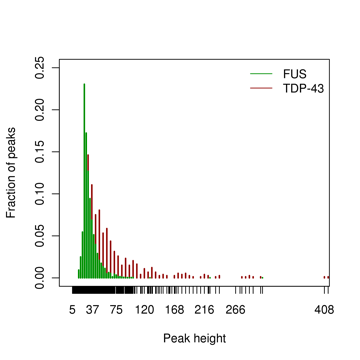
**

**Figure S1I**

**
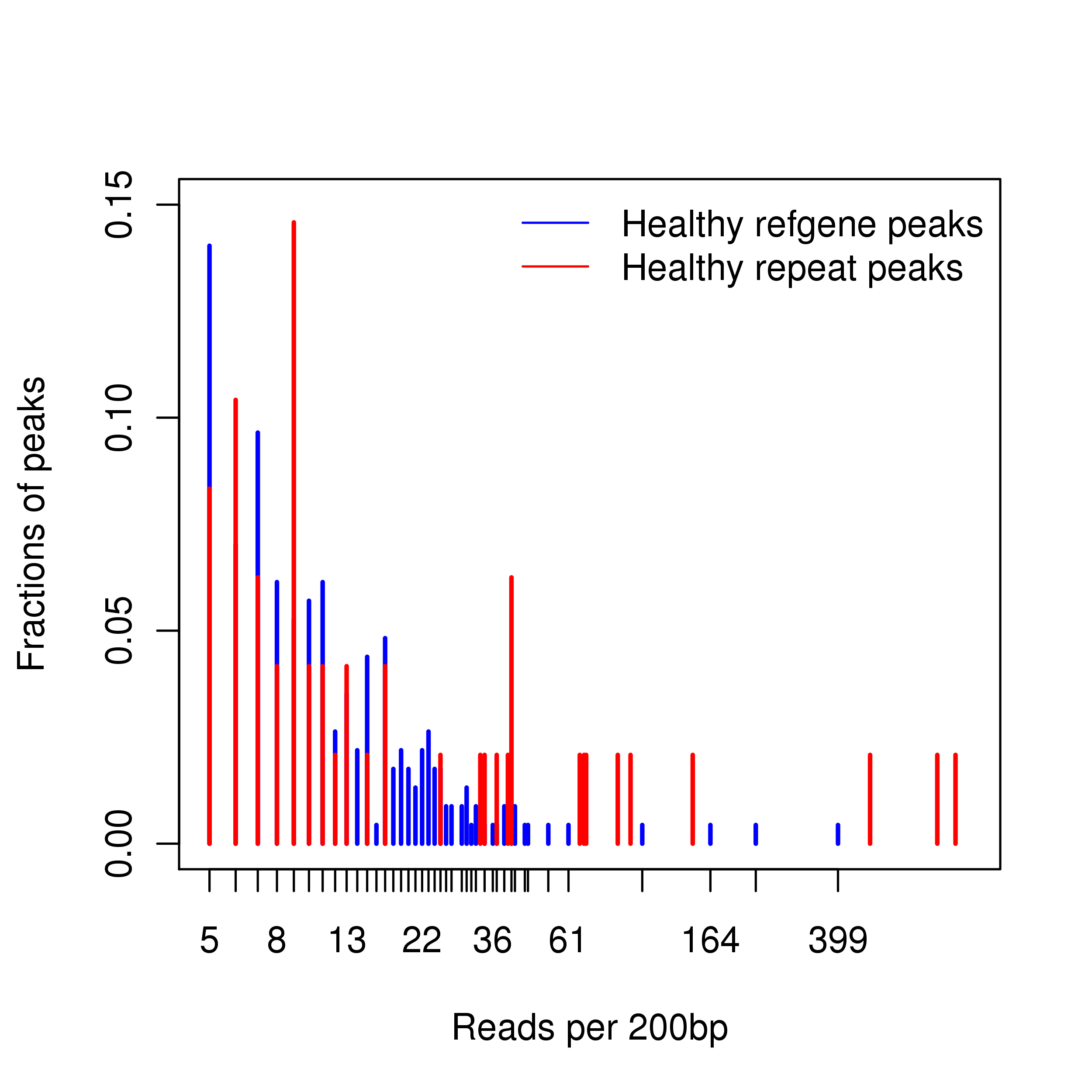
**

**Figure S1J**

**
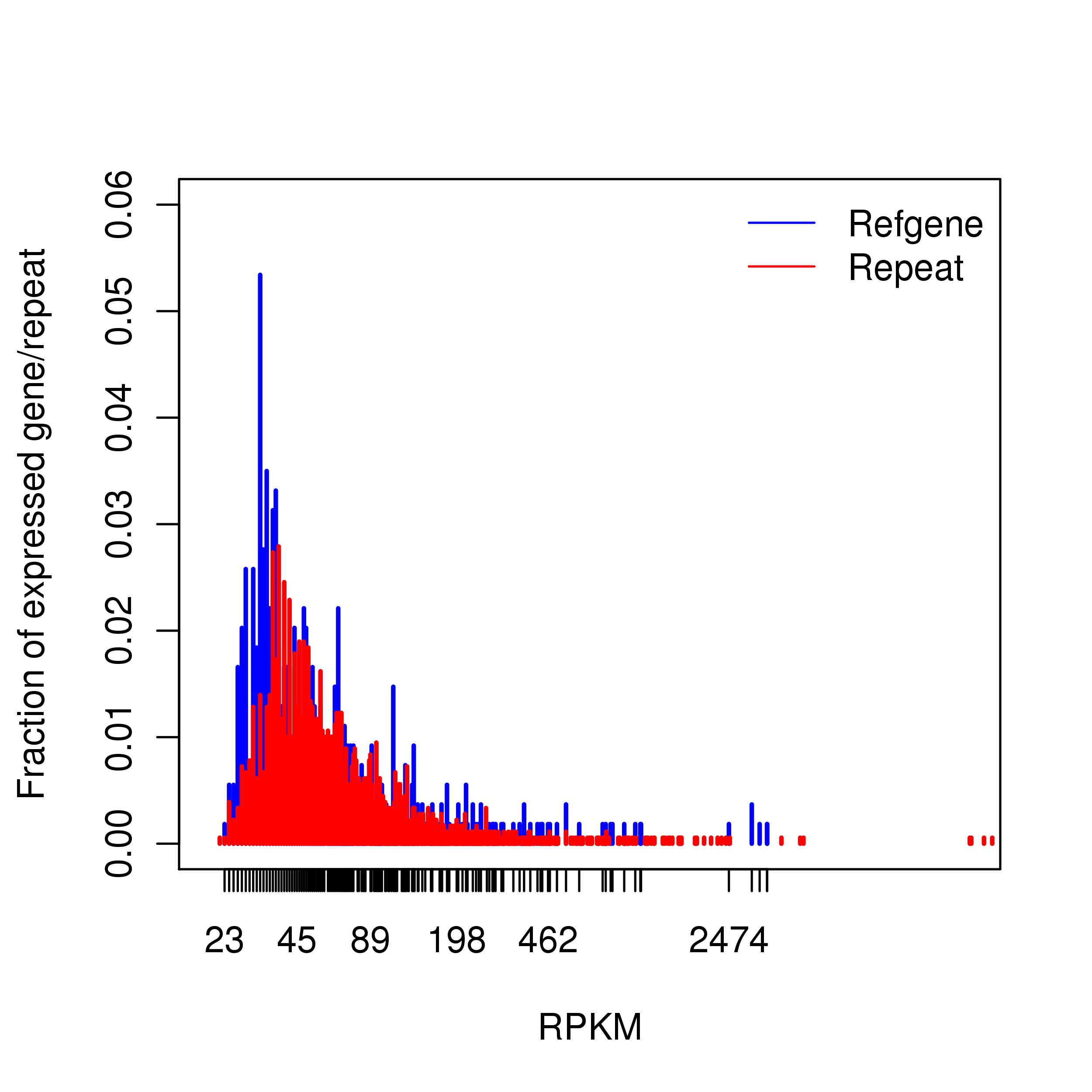
**

**Supplementary References**

1. Polymenidou M, Lagier-Tourenne C, Hutt KR, Huelga SC, Moran J, et al. (2011) Long pre-mRNA depletion and RNA missplicing contribute to neuronal vulnerability from loss of TDP-43. Nature neuroscience 14: 459-468.

2. Shan X, Chiang PM, Price DL, Wong PC (2010) Altered distributions of Gemini of coiled bodies and mitochondria in motor neurons of TDP-43 transgenic mice. Proceedings of the National Academy of Sciences of the United States of America 107: 16325-16330.

3. Tollervey JR, Curk T, Rogelj B, Briese M, Cereda M, et al. (2011) Characterizing the RNA targets and position-dependent splicing regulation by TDP-43. Nature neuroscience 14: 452-458.

4. Sephton CF, Cenik C, Kucukural A, Dammer EB, Cenik B, et al. (2011) Identification of neuronal RNA targets of TDP-43-containing ribonucleoprotein complexes. The Journal of biological chemistry 286: 1204-1215.

5. Da Cruz S, Cleveland DW (2011) Understanding the role of TDP-43 and FUS/TLS in ALS and beyond. Current opinion in neurobiology 21: 904-919.
