# Supplementary material for: Transposable Elements in TDP-43-Mediated Neurodegenerative Disorders
Source: PLoS One. 2012 Sep 5;7(9):e44099. doi: 10.1371/journal.pone.0044099 (PMC3434193; doi:10.1371/journal.pone.0044099)
Supplement: Table S1 — Number of aligned reads for each TDP-43 dataset. Human dataset is from EMBL-EBL ArrayExpress Archive EMTAB-530 and rat dataset is from NCBI GEO DATASET Accession Number: GSE25032. The mouse datasets are from NCBI GEO DATASET Accession Numbers: GSE22351 and GSE27394. (DOCX) [file pone.0044099.s002.docx]

**Supplementary Table S1. Number of aligned reads for each TDP-43 dataset.**

| **Human** | Reads after removing adapters | Uniquely mapped | Unique + multiple alignment | |
| --- | --- | --- | --- | --- |
| Healthy_brain_C23 | 1,821,484 | 29.64% | 41.34% | |
| Healthy_brain_C25 | 3315218 | 62.08% | 87.31% | |
| Healthy_brain_C30 | 2,214,683 | 40.59% | 74.99% | |
| FTLD_TDP_brain_F20 | 2,368,609 | 22.56% | 87.85% | |
| FTLD_TDP_brain_F21 | 1,565,761 | 40.76% | 85.92% | |
| FTLD_TDP_brain_F24 | 1,699,368 | 20.06% | 82.42% | |
|  | | | | |
| **Rat** |  |  |  | |
| Control | 25,626,886 | 15.41% | 84.32% | |
| TDP-43 | 27,291,055 | 42.32% | 82.54% | |
|  |  |  |  | |
| **Mouse** |  |  |  | |
| CLIP-seq (Low Mnase) | 11,422,886 | 46.13% | 74.23% | |
|  |  |  |  | |
| RNA-seq control (rep 1.1) 61 | 16,280,064 | 63.77% | 82.2% |  |
| RNA-seq control (rep1.2) 66 | 21,522,132 | 64.35% | 81.46% |  |
| RNA-seq control (rep2.1) 62 | 17,874,867 | 62.95% | 82.81% |  |
| RNA-seq control (rep2.2) 67 | 23,654,429 | 64.39% | 82.62% |  |
| RNA-seq control (rep2.3) 68 | 23,872,449 | 64.42% | 82.67% | |
| RNA-seq control (rep3.1) 63 | 15,384,509 | 62.99% | 83.29% | |
| RNA-seq control (rep3.2) 69 | 20,366,829 | 64.87% | 83.32% | |
| RNA-seq control (rep3.3) 70 | 20,051,210 | 63.88% | 83.25% | |
| RNA-seq control (rep4.1) 64 | 17,026,225 | 64.74% | 83% | |
| RNA-seq control (rep4.2) 65 | 16,800,571 | 63.45% | 82.5% | |
| RNA-seq control (rep4.3) 71 | 23,401,256 | 62.59% | 82.49% | |
| RNA-seq TDP43  (rep 1.1) 72 | 13,701,340 | 63.72% | 84.87% | |
| RNA-seq TDP43  (rep 1.2) 73 | 13,098,290 | 62.22% | 82.69% | |
| RNA-seq TDP43  (rep 1.3) 76 | 19,583,200 | 64.19% | 82.59% | |
| RNA-seq TDP43  (rep 2.1) 74 | 17,330,196 | 63.42% | 84.79% | |
| RNA-seq TDP43  (rep 2.2) 75 | 15,709,107 | 64.15% | 83.73% |  |
| RNA-seq TDP43  (rep 2.3) 77 | 19,346,369 | 63.35% | 81.35% |  |
| RNA-seq TDP43  (rep 3.1) 78 | 16,524,706 | 64.17% | 81.7% |  |
| RNA-seq TDP43  (rep 3.2) 80 | 23,408,974 | 63.83% | 83.37% | |
| RNA-seq TDP43  (rep 4.1) 79 | 17,291,842 | 64.12% | 83.31% | |
| RNA-seq TDP43  (rep 4.2) 81 | 23,333,313 | 64.34% | 83.34% | |
