# Supplementary material for: Transposable Elements in TDP-43-Mediated Neurodegenerative Disorders
Source: PLoS One. 2012 Sep 5;7(9):e44099. doi: 10.1371/journal.pone.0044099 (PMC3434193; doi:10.1371/journal.pone.0044099)
Supplement: Table S2 — Number of aligned reads for FUS datasets. FUS dataset is from DDBJ Sequence Read Archive (DRA) Accession Number: SRA025082. (DOCX) [file pone.0044099.s003.docx]

**Supplementary Table S2. Number of aligned reads for FUS datasets.**

| **FUS** | Reads after removing adapters and trimming | Uniquely mapped |
| --- | --- | --- |
| Stable | 9,922,520 | 24.24% |
| Inducible 1 | 8,537,179 | 26.56% |
| Inducible 2 | 3,463,758 | 27.26% |
| R521G | 13,325,493 | 23.13% |
| R521H | 11,022,216 | 21.92% |
